# Modifiable predictors of severe heart failure in Morocco: a descriptive study using routinely collected health data

**DOI:** 10.11604/pamj.2019.34.6.17998

**Published:** 2019-09-03

**Authors:** Alpha Kone, Mahamoud Sama Cherif, Dahal Prabin, Shyam Prakash Dumre, Almamy Ibrahim Doumbouya, Diane Fotso Kapche, Facely Camara, Serbout Saousan, Khaddi Sara, Mandiou Diakite, Mohamed Cisse, Leila Azzouzi, Rachida Habbal

**Affiliations:** 1 Department of Cardiology, Ibn Rochd Hospital, Faculty of Medicine and Pharmacy, Hassan II University of Casablanca, Morocco; 2 Faculty of Medicine Pharmacy and Odontostomatology, Gamal Abdel Nasser University of Conakry, Conakry, Guinea; 3 Infectious Diseases Data Observatory, Centre for Tropical Medicine & Global Health, Nuffield Department of Clinical Medicine, University of Oxford, Oxford, UK; 4 Institute of Tropical Medicine (NEKKEN), Nagasaki University, Nagasaki, Japan

**Keywords:** Heart failure, neutrophils-lymphocytes-ratio, hdl, Morocco

## Abstract

**Introduction:**

Heart Failure (HF) is a growing public health concern in Morocco and there is a striking paucity on determinants of severe HF (SHF) in this population. The aim of this study was to identify patients admitted with HF at Ibn Rochd Hospital, Casablanca from 2011 onwards, when electronic record keeping began.

**Methods:**

A total of 105 patients underwent a series of cardiological examinations between July 2011 and January 2014. The New York Heart Association (NYHA) criteria was used to evaluate the severity of HF. Patients with NYHA classification gradings of I and II were defined as having moderate HF (MHF) and those graded as III and IV were defined as having a SHF. Univariable and multivariable risk factors associated with SHF were explored using logistic regression. The results were reported following the RECORD (Reporting of studies Conducted using Observational Routinely-collected Data) statement.

**Results:**

A total of 24 (33%) patients were identified as having a SHF. Four predictors of SHF were identified in univariate analysis: haemoglobin <12g/dL, neutrophil-to-lymphocyte ratio (NLR) >3, mean corpuscular haemoglobin concentration (MCHC) <32 picolitre, and high density lipoprotein (HDL) <0.35 (mmol/L). Only NLR>3 and HDL <0.35 mmol/L remained independent predictors in multivariable analysis. Patients with NLR >3 were at 6-fold increased odds of SHF [adjusted odds ratio (AOR): 6.78, 95% confidence interval (CI): 1.40-32.80, p=0.017], and those with HDL<0.35 (mmol/L) were at 10-fold increased odds of SHF [AOR: 10.11, 95% CI: 2.26-45.27, p=0.002].

**Conclusion:**

The independent biomarkers of SHF identified in this study provide valuable information to ward clinicians in resource-constrained facilities to identify patients vulnerable to developing severe heart complications.

## Introduction

Heart Failure (HF) is the inability of the heart to pump the required amount of blood and oxygen to the peripheral tissues necessary to meet their metabolic demands [1]. It affects at least 26 million people worldwide and therefore exerts a significant and substantial burden to health facilities globally [2]. The prevalence of HF is increasing dramatically with changing age-structure as there is a shift in age-pyramid to an elderly population, a trend also observed in many African countries including Morocco [3]. In Morocco, HF is the cause of quarter of all admissions in the cardiology department nationally [4] and represents a major public health problem [5]. The current gold-standard approach for detecting HF is based on the echocardiographic examinations undertaken in a patient which is the access of which remains limited in constrained settings [6]. In such resource constrained settings, it is highly warranted to have a simple set of prognostic factors derived from routine blood examination, which are cheap, rapid and predictive of heart failure. Such screening measures enables better clinical decision making and thus potentially save lives through prompt and effective case management. Clinical and epidemiological literature has identified various markers associated with heart failure status [7, 8]. These include: old age, female patients, those who are obese with a history of hypertension, diabetes, and non-cardiovascular comorbidities [2, 3], low haemoglobin count [9], increased neutrophils-to-lymphocytes ratio (NLR) [10], ventricular dysfunction [11], and atrial septal aneurysm [12]. However, disease prognosis is multifactorial and represents a complex interplay of social, cultural and genetic factors, and there is a conspicuous paucity of information regarding whether these putative factors are associated with the HF status in Moroccan population. This article aimed to address this gap using data from patients presenting to the cardiology unit of Ibn Rochd hospital in Casablanca in central-western Morocco.

## Methods

**Figure 1:**
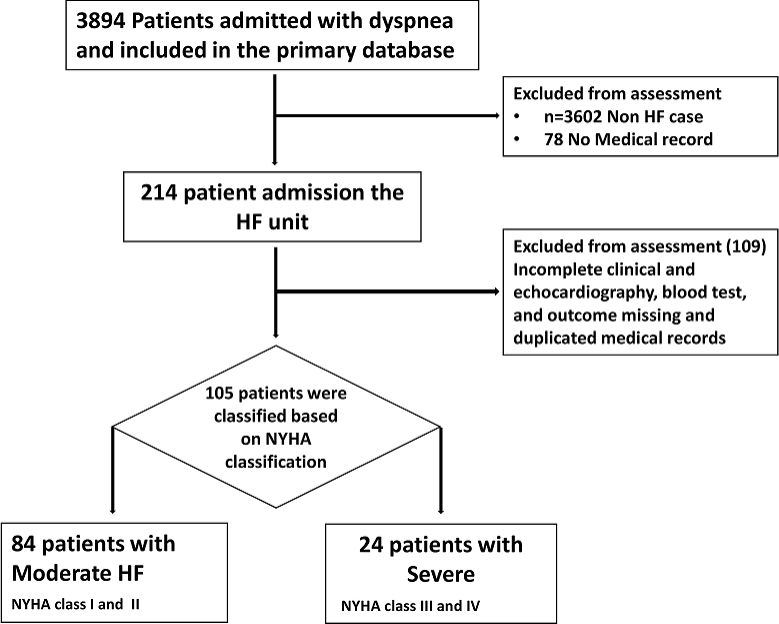
Flow diagram of study design depicting the overview of study population and grading of the study participant into two groups

**Study design:** this was a retrospective descriptive study aimed at characterising the clinical and baseline characteristics of patients with severe heart failure. A clinical audit was carried out to identify patients who were admitted with dyspnoea in the heart failure ward in Casablanca Hospital, Morocco from July 2011 to January 2014 (Figure 1).

**Physical examination:** on admission, patients were asked questions regarding the history of diabetes, high blood pressure, angina pectoris, myocardial infarction (MI), history of medications, and on behavioural habits such as smoking, drinking, and physical exercise. Patients were then asked to be seated for 20 minutes before the measurements of cardiac frequency and blood pressure (systolic and diastolic) were recorded.

**Blood investigation:** on admission, venepuncture was carried out to draw blood samples for examining biochemistry parameters using an automated blood cell counter (Beckman Coulter DxH 800, USA). Fasting blood glucose levels, lipid profiles (high-density lipoprotein (HDL), low-density lipoprotein (LDL), total cholesterol, and triglycerides (TG) levels), aspartate transaminase (AST), alanine transaminase (ALT), gamma-glutamyl transpeptidase (y-GTP), creatinine, uric acid, electrolytes, calcium, phosphate, and total albumin levels were all recorded. Cell counts (neutrophils, red blood cells, and lymphocytes) were obtained.

**Echocardiographic measurements:** a two-dimensional, M-mode pulsed and colour flow doppler echocardiographic examinations were performed using vivid 6 Pro equipped with a 2-5 MHz phased-array transducer (GE Healthcare, Horten, Norway). During echocardiography, a single-lead electrocardiogram was recorded continuously. Atrial septal aneurysm (ASA) was detected by transthoracic echocardiography. M-mode measurements were performed according to the American Society of Echocardiography criteria [13, 14]. Other echocardiographic variables collected include: right atrium surface (RAS), left atrium surface (LAS), tricuspid annular plane systolic excursion (TAPSE), pulmonary arterial blood pressure (PABP), and diameter of inferior vena cava (IVC). The left ventricular ejection fraction (LVEF) was estimated using the modified Simpson's rule as described by Folland *et al.* [15].

**Defining heart failure (HF) status:** the New York Heart Association (NYHA) functional classification [16-18] was used to assign patients into two major categories of HF, moderate HF (Class I and II), and severe HF (Class III and IV). While there exists various classification guidelines on defining severity of HF status, the NYHA functional classification, which relies on the subjective assessment by the ward clinician was used, as this is the most widely used classification rule in routine clinical practice and research [19, 20].

**Statistical analyses:** all statistical analyses was carried out using Stata software, version 15.0 [21]. The primary endpoint used in this analysis was severe HF status as gauged by the NYHA functional classification criteria, that is, those who were graded class III and IV. The baseline characteristics of the patients were presented as median and interquartile range (IQR) for continuous variables, and as proportions for categorical variables. In unadjusted analysis, the comparisons of the baseline characteristics between moderate and severe HF groups were carried out by using Wilcoxon rank sum test for non-normally distributed continuous variables, and Chi-squared test (χ^2^) for categorical variables. All statistical tests were considered as being significant if p-value is <0.05. Univariable and multivariable analysis of risk factors associated with severe HF status was conducted using a logistic regression model. Neutrophils-to-lymphocytes (NLR) ratio (NLR) was categorised as above or below 3.0, a threshold which has been previously shown to have high sensitivity and specificity [10]. All variables significant at 5% level in univariable analysis were included in the multivariable analysis. Inclusion of covariates in the final model was based on their effect on model coefficients and the degree to which they improved the overall model based on a likelihood ratio test. The robustness of the point estimates of the regression coefficients in the final multivariable model was assessed using jacknified procedure obtained by removing one observation at a time and the result was summarised as coefficient of variation (CV).

In addition, 1,000 bootstrap resamples of the same size as the original data were drawn and the distribution of regression coefficients were plotted and used to estimate the confidence interval for the respective coefficients. The goodness of fit of the final fitted logistic regression model was evaluated using the Hosmer-Lemeshow test using estat gof command in stata. [22, 23]. The population attributable risks (PARs) for the variables in the final multivariable model for severe HF were calculated based on the prevalence of the risk factors in the study population and its associated relative risk (adjusted odds ratio(OR)) [24]. The overall PAR (for a combination of risk factors), which is non-additive, was calculated as 1-[(1-PAR1)×(1-PAR2)×..×(1-PARn)]. Missing data were handled using multiple imputation approach-estimates and standard errors were pooled across the imputations using Rubin's combination rules. The number of imputations (m=50) were selected following the recommendation that m should be at least equal to the percentage of missing cases when the fraction of missing information is less than 50% [25]. The results were reported following the RECORD (Reporting of studies Conducted using Observational Routinely-collected Data) statement [26].

## Results

**Figure 2:**
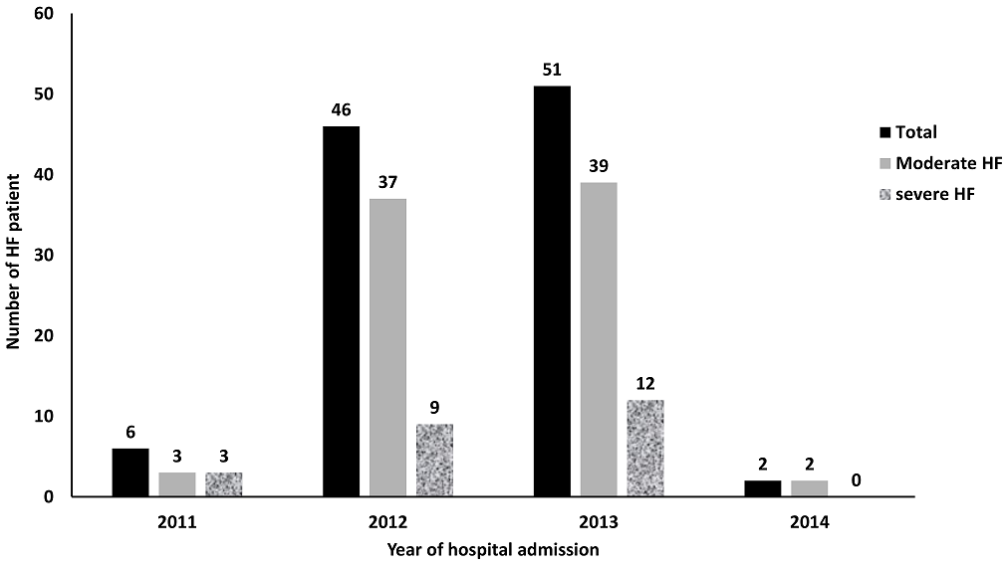
Distribution of HF patient by year and by group of patients (moderate and severe HF)

**Baseline characteristics and clinical grading:** a total of 105 patients who were admitted with dyspnea in the Ibn Rochd hospital in Casablanca, Morocco between July 2011 and January 2014 were included in the study (Figure 1, Figure 2). A total of 24 patients (23%) were classified as severe cases of heart failure; the baseline characteristics of the patients are described in Table 1.

**Table 1:** Baseline characteristic study participants (N= 105 patients)

Variables	Moderate /severe* Or total HF number	Moderate HF, n (%)	Severe HF, n (%)	P-value
**Demographic**				
Age (years)*	81/24	64.8 (55.5-72.6)	58.4 (52.3-67.6)	0.112*
Male (n)	68	56 (69.1)	12 (50.0)	0.089
**Risk factors (n)**				
Presence of HTA	37	28 (34.6)	9 (37.5)	0.792
Presence of diabetes	28	21 (25.9)	7 (29.2)	0.753
Presence of dyslipidaemia	10	6 (7.4)	4 (16.7)	0.231
Tobacco use	30	25 (30.9)	5 (20.8)	0.339
Menopause	16	11(13.6)	5 (20.8)	0.384
Lack of physical activities	22	17 (21.0)	5 (20.8)	0.987
History of stroke	4	3 (3.7)	1 (4.2)	1.000
History of myocardial infarction (MI)	22	18 (22.2)	4 (16.6)	0.557
Chronic bronchopneumopathy	5	3 (3.7)	2 (8.3)	0.321
Clinical signs*				
Heart beats (/min)	80/24	83.5 (69.3-94.8)	81.0 (66.3-102.3)	0.835
Blood pressure systolic (/mmHg)	77/24	120 (110-147.5)	180 (101-129)	0.073
Blood pressure diastolic (/mmHg)	77/24	72.0 (66.0-84.0)	70.0(60.0-80.0)	0.175
ECG*				
PR interval (seconds)	62/22	0.16 (0.12-0.16)	0.08 (0.12-0.20)	0.110
QRS (seconds)	74/22	0.08 (0.06-0.10)	0.08 (0.08-0.12)	1.000
Echocardiogram*				
Left Atrium Surface (LAS) (cm^2^)	17/07	24.0 (30.0-27.5)	32.0 (16.0-46.0)	0.309
Right Atrium Surface (RAS) (cm^2^)	15/02	13.0 (11.5-20.0)	12.5 (7.0-12.90)	0.618
TAPSE (mm)	20/05	18.0 (12.75-24.0)	20.0 (12.5-23.0)	0.973
PABP (mmHg)	44/13	39.0 (25.0-55.0)	50.0 (42.5-62.0)	0.053
IVC#0 (mm)	26/06	24.5 (14-30.0)	17 (15.0-19.0)	0.225
TG#0	61/18	1.70 (0.73-1.48)	1.25 (0.70-1.45)	0.799
LVEF percentage	81/24	35.0 (30.0-44.0)	32.5 (23.75-40.0)	0.110
Blood parameters*				
Lymphocytes (/mm^3^)	76/24	1950 (1532-2460)	1520 (1220-2157)	0.034
Neutrophils (/mm^3^)	76/23	4645 (3487-5620)	4850 (4180-6880)	0.161
Platelets (10^2^/mm^3^)	76/23	2425 (1997-2937)	2350 (1510-2720)	0.622
Neutrophil Lymphocytes Ratio (NLR)	76/23	2.24 (1.65-3.08)	3.24 (1.92-6.10)	0.010
Neutrophil Platelet Ratio (NPR)	76/22	0.018 (0.01-0.02)	0.021 (0.01-0.03)	0.100
Hemoglobin (g/dL)	81/23	12.7 (11.9-13.8)	11.6 (11.0-12.9)	0.038
MCV (femtolitre)	81/23	84.4 (81.0-87.45)	84.6 (80.2-88.3)	0.891
MCHC (Pico litre)	87/23	33.3 (32.1-34.05)	32.7 (31.3-33.5)	0.009
Haematocrit percentage	76/23	38.3 (36.0-41.8)	36.5 (32.7-40.5)	0.378
Blood sodium (me/L)	65/22	139 (136-142)	137.5 (134-140)	0.140
Blood potassium (me/L)	62/21	4.92 (4.37-5.36)	5.31 (4.73-5.94)	0.371
AST (UI/L)	67/20	23.0 (18.0-32.0)	21.0 (17.0-29.7)	0.443
ALT (UI/L)	68/20	18.0 (13.0-33.75)	15.0 (9.5-17.75)	0.030
Prothrombin ratio	26/04	70.5 (30.0-95.7)	38.5 (31.0-55.0)	0.245
Uric acid (mg/L)	21/12	65.1 (50.6-84.25)	77.8 (63.3-114.1)	0.190
INR of Prothrombin	16/04	1.32 (1.07-2.48)	2.1 (1.72-2.43)	0.299
Total cholesterol (moll/L)	52/18	1.70 (1.43-2.04)	1.36 (1.15-1.72)	0.024
HDL (mol/L)	58/18	0.50 (0.39-0.62)	0.36 (0.30-0.4)	<0.001
LDL (mol/L)	54/18	1.0 (0.79-1.30)	0.97 (0.73-1.2)	0.482
Urea (g/L)	73/24	0.45 (0.29-0.64)	0.58 (0.41-1.15)	0.025
Creatinine (mg/L)	68/24	10.9 (8.72-14.07)	12.6 (9.0-17.4)	0.174
Creatinine clearance (mL/min)	65/24	64.0 (39.0-86.00)	51.5 (35.5-77.4)	0.432
Blood glucose (g/L)	67/22	0.93 (0.76-1.28)	0.93 (0.78-1.3)	0.581
Fibrinogen (mg/ld)	21/06	3.52 (2.80-4.52)	4.21 (2.63-5.7)	0.299
Albumin (mg/L)	07/04	46.8 (43.5-49.6)	42.3 (34.7-43.7)	0.042

* These quantitative variables among moderate and severe heart failure (HF) groups are compared by Wilcoxon Rank Sum test and expressed as median (and IQR). All other categorical variables are expressed as proportions (number (%)) and compared by Chi-Squared test or Fisher’s exact test (when the expected cell count was < 5). A *p*-value < 0.05 was considered as being statistically significant. Abbreviations: Electrocardiogram (ECG); Duration rom the onset of the P wave to the start of the QRS complex (PR); Duration of ventricular depolarisation (QRS); Tricuspid annular plane systolic excursion (TAPSE); Pulmonary Arterial Blood Pressure (PABP); Inferior Vena Cava (IVC); Left ventricular ejection fraction (LVEF); International normalized ratio (INR) of prothrombin time of blood coagulation; Mean corpuscular volume (MCV); Mean corpuscular haemoglobin concentration (MCHC); Aspartate aminotransferase (AST); Alanine aminotransferase (ALT); High density lipoprotein (HDL) and Low density lipoprotein (LDL)

**Figure 3:**
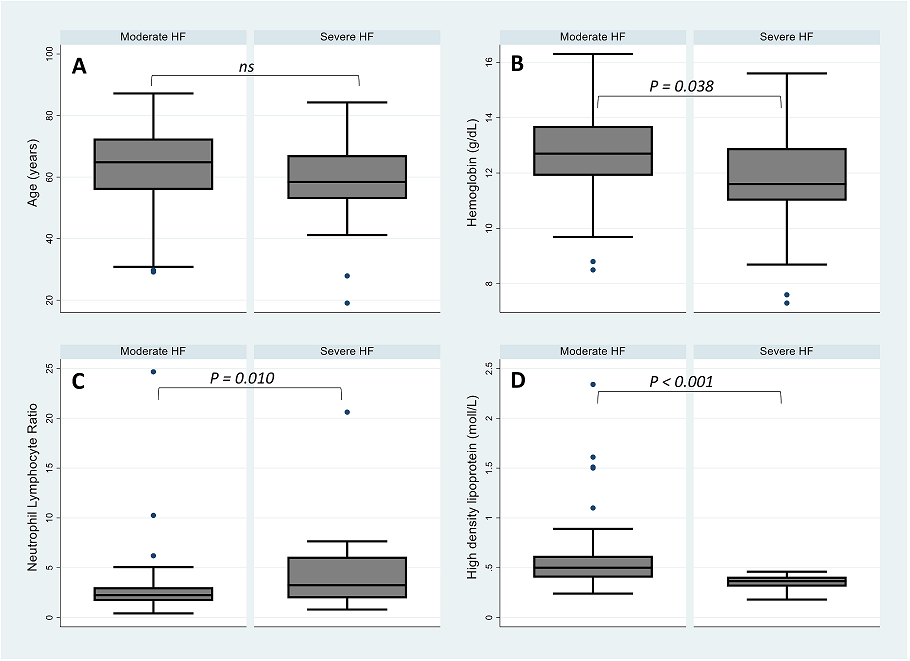
Comparison of distribution of age (A); hemoglobin levels (B); neutrophil lymphocyte ratio (C); and HDL levels (D); measured on admission between moderate and severe HF patient groups

**Figure 4:**
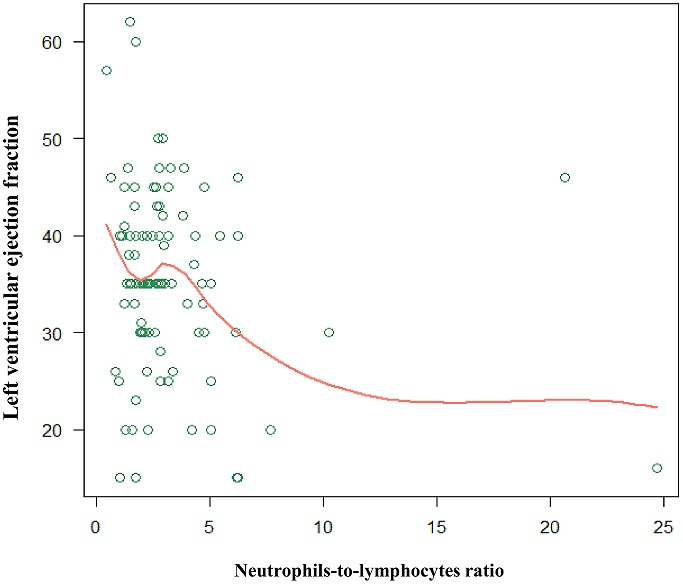
Relationship between left ventricular ejection fraction and neutrophil to lymphocytes ratio. Legend: Pearson's correlation coefficient. -0.169, P-value: 0.092. The red line depicts the lowess (locally weighted scatterplot smoothing) smoother

**Summary statistics in patients with severe and moderate heart failure:** the median age was 64.8 years [inter-quartile range (IQR): 55.5-72.6] in patients classified as moderate HF and 58.4 [IQR: 52.3-67.84] for those who were classified as having severe HF (P-value=0.112) (Table 1, Figure 3). The proportion of risk factors such as diabetes, hypertension, and dyslipidaemia were not different between two groups of patients. Similarly, patient's history of tobacco use, lack of physical activity, cerebral vascular accident, chronic bronchopneumopathy and asthma revealed no difference between the two groups (Table 1). Patients with severe HF were found to be associated with low high-density lipoprotein (HDL), total cholesterol (TC), alanine aminotransferase (ALT), mean corpuscular haemoglobin concentration (MCHC), haemoglobin (HB) and lymphocyte counts, while an increased NLR was significantly associated with the severe HF (Table 1, Figure 4).

**Figure 5:**
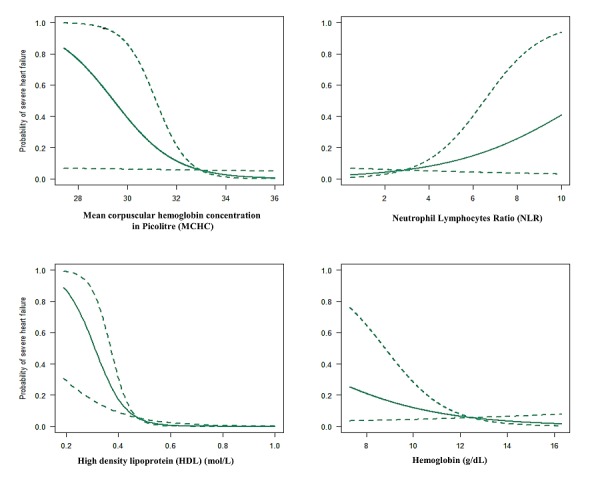
The predicted probability of severe heart failure based on admission characteristics, (A) haemoglobin levels; (B) neutrophil-to-lymphocyte ratio (NLR); (C) mean corpuscular haemoglobin concentration (MCHC); (D) HDL levels (HDL)

**Logistic regression for identifying risk factors for severe heart failure:** in univariable analysis, there were four factors which were associated with severe HF: haemoglobin <12g/dL [OR: 2.68, 95% CI: 1.04-6.85, p=0.039]; NLR >3 [OR: 3.33, 95% CI: 1.29-8.52, p=0.012]; MCHC <32 pL [OR: 2.83, 95% CI: 1.01-7.76, p=0.043]; and high-density lipoprotein (HDL) levels <0.35 mmol/L [OR: 8.48, 95% CI: 2.36-33.56, p=0.001] (Table 2). The probability of severe heart failure when the HDL, MCHC, haemoglobin and NLR are present are summarized in Figure 5. In a multivariable analysis which included all the four predictors identified in the univariable analysis, only two variables were independent predictors of severe HF: NLR greater than 3 [Adjusted OR: 6.78, 95% CI: 1.40-32.80, p=0.017] and HDL <0.35 mmol/L [Adjusted OR: 10.11, 95% CI: 2.26-45.27, p=0.002]. Overall, the model accounted for 93.5% of all the severe HF failure cases, with an NLR>3 accounting for 65.0% (Table 2).

**Table 2:** Univariable and multivariable logistic regression analysis for severity of HF

		Univariable (N=105)			Multivariable analysis (N=70) b				
	N(n)	Unadjusted OR	95% CI	P-value	Adjusted OR	(95% CI)	P-value	Prevalence (%)	PAR c (%)
**Demographic**									
Age (/year)	105(24)	1.03	0.99-1.06	0.128					
Sex (reference: Male)	68(12)	0.45	0.18-1.13	0.089					
**History**									
History of hypertension	37(9)	1.14	0.44-2.92	0.792					
History of diabetes	28(7)	1.18	0.43-3.23	0.753					
History of tobacco use	30(5)	0.59	0.19-1.76	0.343					
Menopause	16(5)	1.66	0.52-5.41	0.389					
Lack of physical activities	22(5)	0.99	0.32-3.04	0.987					
History of myocardial infarction	22(4)	0.70	0.21-2.31	0.558					
**Physical examination**									
Heart beat > 100 (/min)	104(24)	1.65	0.58-4.64	0.346					
Systolic blood pressure > 150 mmHg	101(24)	0.44	0.11-1.62	0.216					
Diastolic blood pressure > 90 mmHg	101(24)	0.76	0.22-2.54	0.660					
**Echocardiogram**									
PAPS > 30	57(13)	6.60	0.80-56.4	0.085					
SOG > 30	24(7)	6.22	0.88-43.7	0.066					
TAPSE £ 18	25(5)	1.23	0.16-9.01	0.840					
VCI ≥ 20	32(6)	3.61	0.77-16.7	0.101					
**Blood parameters**									
Haemoglobin < 12 (g/dL)	104(24)	2.68	1.04-6.85	0.039	3.54d	0.84-14.97	0.086	34	45.32
MCV < 80 (femtolitre)	85(18)	0.75	0.24-2.37	0.626					
MCHC £ 32 (picolitre)	102(22)	2.83	1.01-7.76	0.043	3.43 d	0.70-16.87	0.128	24	35.95
NLR > 3	99(32)	3.33	1.29- 8.52	0.012	6.78 d	1.40-32.80	0.017	32	65.09
Hyperkalemia > 5.1 (mEq/L)	80(62)	1.40	0.55-3.56	0.479					
LDL > 1.5 (mmol/L)	70(64)	1.67	0.18-15.4	0.652					
HDL £ 0.35 (mmol/L)	76(13)	8.48	2.36-33.56	0.001	10.11 d	2.26-45.27	0.002	13	60.88
Glycaemia >1.24 (g/L)	79(13)	1.10	0.37-3.27	0.860					
AST > 40 (UI/L)	87(10)	0.82	0.16-4.21	0.812					
ALT > 45 (UI/L)	88(8)	0.46	0.05-3.97	0.476					

^a^ Number of patients (N) for each variable (*n*=number of severe HF)

^b^ Hosmer-Lemeshow goodness of fit for the final multivariable model *P*= 0.84

^c^ Overall Population Attributable Risk (PAR) for the final multivariable model was 93.5%

^d^ The coefficient of variation (CV) for the variables in the final multivariable model obtained by removing one observation at a time were: 5.1%, 6.6%, 3.8% and 7.7% for NLR > 3, haemoglobin < 12 g/dL, HDL levels £ 0.35 and MCHC £ 32 respectively. The adjusted odds ratio from a multivariable analysis when these variables were fitted as continuous predictors are: haemoglobin [AOR: 0.68, 95% CI: 0.45-1.01, *p*=0.061]; NLR [AOR: 1.29, 95% CI: 0.87-1.89, *p*=0.207] and HDL [Adjusted OR: 2 e-08, 95% CI: 5 e-13-1.5 e-03, *p*=0.002].

OR= Odds Ratio; CI = Confidence Interval; BPCO = chronic bronchopneumopathy; PAPS = Pulmonary artery systolic pressure; LAS= Left auricular surface; Tricuspid annular plane systolic excursion (TAPSE); Diameter of inferior vena cava (IVC); Mean corpuscular volume (MCV); Mean corpuscular haemoglobin concentration (MCHC); Neutrophil-to-lymphocytes ratio (NLR); Low density lipoprotein (LDL); High density lipoprotein (HDL); Aspartate aminotransferase (AST); Alanine aminotransferase (ALT).

**Sensitivity analyses:** the results of the sensitivity analyses carried out are presented in Table 3. There were four observations with unusual values of NLR ratio (Figure 3); the exclusion of which from the analysis led to the conclusion remaining unchanged (Table 3). The coefficient of variation for the variables in the final multivariable model obtained from jack-knifing procedure were all <10% suggesting that the regression estimates weren't vulnerable to any particular influential observation. Results from multiple imputation analysis to handle missing variables were similar to the ones obtained from the final multivariable model suggesting that missing observations had little impact on the estimated regression coefficients. Finally, the adjusted odds ratio derived from 1,000 bootstrap resamples drawn from the data were similar to the point estimates from the final regression model, again suggesting that the derived estimates were robust (Data not shown).

**Table 3:** Sensitivity analyses of NLR Outliers and the effect on final prediction model (Continuous)

	Final multivariable model with outliers		Final multivariable model without outliers	
Variable	Adjusted odds ratio [95% confidence interval]	*P-value*	Adjusted odds ratio [95% confidence interval]	*P-value*
Hb < 12 g/dL	0.71 [0.46-1.09]	0.126	0.60[0.35-1.03]	0.063
HDL< 0.35 (mmol/L)	3.15e-08 [1.24e-12-0.000798]	0.001	1.69e-09[5.61e-15-0.0005063]	0.002
NLR>3	1.41 [0.92-2.17]	0.117	2.35 [1.22-4.53]	0.010
MCHC <32 (picolitre)	0.44 [0.21-0.96]	0.040	0.33 [0.12-0.89]	0.029

Hb= Haemoglobin (g/dL); HDL = High density lipoproteins; NLR = Neutrophils-to-Lymphocytes Ratio; MCHC= Mean corpuscular haemoglobin concentration

## Discussion

We carried out a MEDLINE search using the combination of terms “Morocco” AND “Heart Failure” which identified 40 records, none of which explored factors associated with heart failure. To our knowledge, this is the first study reporting predictors of severe HF in Moroccan patients and we report two independent predictors of severe heart failure using data from 105 patients admitted to the cardiology unit of Ibn Rochd hospital in Casablanca.

First, patients with neutrophil-to-lymphocytes (NLR) ratio greater than 3 were associated with 6.7-fold increased odds of experiencing severe HF. This could be explained by the fact that, an inverse correlation was observed between NLR and left ventricular ejection fraction (LVEF), which is a known marker of heart failure [10]. In our dataset, every unit increase in NLR was associated with a drop in LVEF by 20.58 units when adjusted for age, gender, haemoglobin and MCHC levels (P-value= 0.0063) (Figure 4). Our result corroborates well with an earlier report which demonstrated that NLR >3 was a predictor of heart failure [10] and is consistent with several other studies have shown an increased NLR is associated with cardiovascular diseases [12, 27], adverse cardiac events among diabetic population [28], and fatal outcomes [10, 29, 30]. White blood cells (WBCs) including lymphocytes and neutrophils produces cytokines (such as TNF-α, IFN-Υ, IL-1β, IL-6, IL-17, and IL-18) in responses to stress, tissue injuries or inflammation. These cytokines can induce cardio myocyte, hypertrophy, apoptosis, fibrosis, and ultimately lead to adverse cardiac events [31, 32]. Secondly, our study showed that the high-density lipoprotein levels were significantly reduced in patients with severe HF. This is consistent with the fact that low levels of HDL cholesterol increases the risk of heart disease by decreasing the elimination of LDL cholesterol that is known to contribute to plaque in the cardiac arteries (atherosclerosis) which raises the risk for heart attack and stroke [33].

There were several limitations to this analysis. First, we were limited to the retrospective nature of the study carried out using data collected in routine hospital settings. As with all such observational studies, the analysis presented in this report are subject to confounding bias and hence these results should be interpreted with these caveats in consideration. Nonetheless, these findings provide important and valuable information for the clinical practitioners working in resource-constrained settings. Future confirmatory work should focus on the prospective and rigorous evaluation of the biomarkers which were correlated with severe heart failure in this study. Secondly, our analysis was restricted to a relatively small sample of population and hence posed additional difficulties in regression analysis. The estimated confidence intervals for the regression parameters were wide, and hence the effect size and standard errors presented in this reported might have been inflated. The result of the additional sensitivity analyses confirmed that the results of the estimated regression coefficients were robust (Table 3). Thirdly, as with most data collected in routine settings in clinical management of patients, data on a high proportion of observations for HDL were missing (27.6%, 29/105). Multiple imputation (MI) was used to handle these missing observations and the results obtained from the MI analysis were similar to the estimates obtained from final multivariable model, thus reassuring that the estimates weren't affected by missing observations. Finally, we have used the NYHA functional classification for defining heart failure as this is the most commonly used grading system in clinical practice. Hence, the results reported in our article might not be generalizable to other classification measures. [20, 34].

Our results can have several implications and can help ward clinicians in effective and optimal case management. First, some of the risk factors of severe heart failure identified in this study are amenable to behavioural and dietary changes and thus are preventable. Morocco is undergoing an economic transition, especially Casablanca which represents an urban and affluent area of the country. As the working practice and lifestyle changes with more sedentary lifestyle (of working in an office)-healthy eating and regular exercised should be encouraged, which will eventually reduce the risk associated with some of the modifiable risk factors identified (such high HDL levels) [35]. For example, anaemic patients, who were associated with an increased risk in our dataset (although it didn't reach statistical significance), might be given iron and haematinics supplements, and HDL can be acquired as a part of regular diet. Secondly, our study supports the utility of inflammation status as gauged by elevated neutrophils-lymphocytes (NLR) ratio which reflects the dynamic response of the immune system during inflammation [36] an independent predictor of severe heart failure. Inflammation plays a potential role in the disease pathogenesis [7], and hence in theory, this is ideally suited for studying the disease prognosis. We found that NLR was correlated with left ventricular ejection fraction, a key metric used for defining heart failure (Figure 4). In resource-constrained settings with a limited or no access to echocardiographs, NLR ratio can be used as a biomarker to aid clinical decision making for prompting patients to intensive level of care.

## Conclusion

In conclusion, this study reported that an elevated neutrophil-to-lymphocytes ratio, and high-density-lipoprotein level were independent risk factors for a severe HF in Moroccan patients. In resource-limited health facilities like ours, patients who present with a combination of these putative factors should be provided an elevated care without delay.

### What is known about this topic

- HF is the cause of quarter of all admissions in the cardiology department;
- The echocardiographic examinations is gold-standard approach for detecting HF.

### What this study adds

- An elevated neutrophil-to-lymphocytes ratio, and high-density-lipoprotein level are Simple biomarker for severe heart failure;
- These markers are important for resource-limited health facilities.

## Competing interests

The authors declare no competing interests.
